# Terbinafine in acrylic polymer for the treatment of onychomycosis in hemodialysis patients: a phase II clinical trial

**DOI:** 10.3389/fmed.2024.1417985

**Published:** 2024-11-26

**Authors:** Jeanne Marie Queiroz Borges Bersano, Matheus Gobbo Cordeiro, Juliana Mozer Sciani, Iara Lúcia Tescarollo, Fernando Augusto Lima Marson

**Affiliations:** ^1^Laboratory of Molecular Biology and Genetics, Health Sciences Postgraduate Program, São Francisco University, Bragança Paulista, Brazil; ^2^Laboratory of Clinical and Molecular Microbiology, Health Sciences Postgraduate Program, São Francisco University, Bragança Paulista, Brazil; ^3^LunGuardian Research Group – Epidemiology of Respiratory and Infectious Diseases, Health Sciences Postgraduate Program, São Francisco University, Bragança Paulista, Brazil; ^4^São Francisco University Natural Products Laboratory, Health Sciences Postgraduate Program, São Francisco University, Bragança Paulista, Brazil; ^5^Health Sciences Postgraduate Program, São Francisco University, Bragança Paulista, Brazil

**Keywords:** antifungal, clinical trial, kidney dialysis, microbiology, nail plate debridement, onychomycosis

## Abstract

**Introduction:**

Onychomycosis is a nail infection caused by dermatophyte fungi, non-dermatophyte fungi, and yeast. Patients with chronic kidney disease on dialysis are part of the population that presents higher rates of this disease, mainly due to immunosuppression. Among patients with chronic kidney disease on dialysis, the treatment of onychomycosis is complex, mainly due to the limitations imposed by comorbidities. In this context, the study evaluated the safety and potential efficacy of a treatment that combines nail debridement with the use of acrylic gel nails carrying terbinafine at a concentration of 2%.

**Methods:**

Patients from the Hemodialysis Center of the São Francisco de Assis University Hospital in Bragança Paulista, São Paulo, Brazil were included. Those had hallux onychomycosis with clinical forms whose treatment involved the need for nail debridement. After the debridement procedure, a nail prosthesis made with acrylic reconstruction gel and 2% terbinafine was applied. The procedure was renewed every 2 weeks (~14 days) for 11 months. The evolution was monitored with measurements of the normal-appearing nail plate and photographs. Direct mycological examination and fungal culture were performed at the beginning of the study and 30 days after applications were interrupted. Assessment of clinical response, clinical cure, mycological cure, and complete cure was performed at the end of the study. All participants answered a questionnaire about their perception of the treatment.

**Results:**

Out of the 155 patients on hemodialysis, 64/155 (41.3%) individuals were identified with symptoms suggestive of onychomycosis in the halluces after clinical analysis. Among them, 35/64 (54.7%) individuals presented a positive direct mycological examination and underwent fungal culture to identify the etiological agent. In this group of patients, 24/35 (68.6%) individuals who presented clinical forms whose treatment involved the need for nail debridement were selected. Only 15/24 (62.5%) individuals completed the study. Among the study participants, 5/15 (33.3%) still presented positive fungal culture in the presence of a negative direct mycological examination and 1/15 (6.7%) presented a positive direct mycological examination, but with a negative culture. Among those with a positive fungal culture, 3/15 (20.0%) participants presented microorganisms different from those isolated in the initial exams. Regarding cure, 5/15 (33.3%) participants showed a clinical response, 4/15 (26.7%) clinical cure, and 3/15 (20.0%) complete cure. No patient presented an allergic reaction or local irritation caused by the material used in the treatment. There were accidental superficial ulcerations caused by the electric sandpaper; however, no wound developed secondary infection. No participant reported discomfort due to the nail prosthesis use, 3/15 (20.0%) reported a feeling of discomfort caused by the vibration of the electric file and 12/15 (80.0%) reported the perception that their nails had a better appearance during treatment with nail prosthesis made with acrylic reconstruction gel and 2% terbinafine.

**Conclusion:**

The application of 2% terbinafine in acrylic reconstruction gel for the manufacture of nail prostheses applied after debridement of moderate and severe forms of onychomycosis showed low efficacy as an isolated treatment in patients on dialysis due to chronic kidney disease. On the other hand, most patients had a good perception of the appearance of their nails during treatment, even when it did not result in apparent clinical improvement or complete cure.

## Graphical abstract

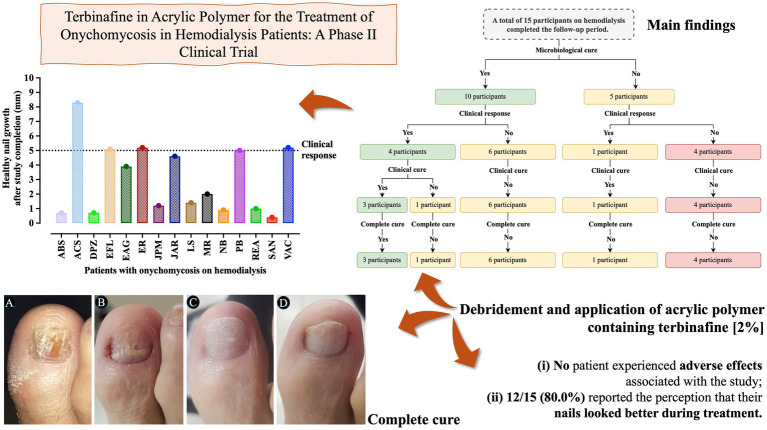

The images show one severe onychomycosis case in which a complete cure was observed after the intervention period carried out with nail debridement followed by the application of acrylic polymer with 2% terbinafine. In this case, we can see as follows: (A) the appearance of the hallux nail at the time of the initial evaluation – study inclusion period, when the direct mycological and fungal culture exams were carried out; (B) the appearance of the hallux nail after the first debridement and cleaning. The ungual plate debridement was carried out only in the cases indicated such as the presence of dermatophytoma, +2 mm thick subungual keratosis, or prolonged onycholysis; (C) the nail appearance during the intervention period. It seems relevant to observe that the polymerized acrylic gel with 2% terbinafine guaranteed the nail healthy appearance. (D) Nail appearance after 11 months of treatment with the polymerized acrylic gel with 2% terbinafine. The outcomes were evaluated after the intervention period. The intervention was carried out with the nail debridement followed by the application of acrylic polymer with 2% terbinafine. Cure criteria were set based on the clinical progression and the laboratory exams and were designed as follows: (clinical response) healthy nail 5 mm growth, (mycological cure) negative direct mycological and fungal culture exams, (clinical cure) nail with a completely normal appearance, and (complete cure) presence of clinical and mycological cure.

## 1 Introduction

Onychomycosis is a term that encompasses all forms of fungal infections in the nails and presents signs and symptoms similar to other pathological conditions, requiring, for this reason, a complementary examination to detect the fungus presence and establish a definitive diagnosis (1, 2). Due to the slow growth of the nail plate, the treatment is long lasting and expensive (1, 2). The most common etiological agents are dermatophyte fungi (~70% of cases), mainly from the genus *Trichophyton*, which has two species of greater epidemiological importance – *T. rubrum* and *T. mentagrophytes* (3–6). Non-dermatophyte filamentous fungi are responsible for ~20% of the cases, and the most common species are *Scopulariopsis brevicaulis*, *Aspergillus* spp., *Acremonium* spp., *Fusarium* spp., *Alternaria alternate* and *Neoscytalidium* spp. (3–6). Also, the yeasts of the genus *Candida* are responsible for 10–20% of this disease cases (3–6). The clinical forms of onychomycosis are classified according to the pattern of nail invasion, as white superficial, distal/lateral subungual, proximal subungual, and endonyx (1, 2, 4). There is also a mixed pattern when more than one clinical type is found in the same nail (1, 2, 4, 6, 7). Finally, the total dystrophic pattern occurs when the nail plate is seen to be extensively and seriously destroyed and dystrophic, corresponding to the advanced state of the other clinical forms (1, 2, 4, 6, 7). Commonly, toe nails are more frequently affected than finger nails (8).

The onychomycosis treatment is hampered by the impermeable nature of the nail, a characteristic ascribed to disulfide bonds and hydrogen bonds within the keratin network, making the nail plate a difficult biological barrier to overcome (9, 10). Fungi have the ability to form a biofilm on the nail plate, leading to the development of resistance against antifungal agents and providing protection against the immune system (11, 12). Therefore, personalized treatment should be administered based on the nail involvement extent, infecting organism, presence of comorbidities, and the simultaneous use of other medications, due to the risk of drug interactions and adverse effects (1, 13–15).

Systemic treatment of onychomycosis is recommended as an initial approach when the nail matrix is involved, if three or more nails are simultaneously affected, in the presence of dermatophytoma, subungual hyperkeratosis greater than 2 mm, and in the total dystrophic form (2, 13, 15). Terbinafine is widely known as the most efficient systemic medication to treat onychomycosis (16). This medication inhibits the cytochrome P450 (CYP450) 2D6 (CYP2D6) system pathways, with relatively low risks of drug interactions and generally negligible adverse effects (16–18). On the other hand, itraconazole, the second most effective systemic drug, inhibits the CYP450 P3A4 (CYP3A4) system and has the potential to interfere with the metabolism of several other medications, such as statins, immunosuppressants, anticonvulsants, coumarins and antiretrovirals (19–21). Moreover, itraconazole has the potential to cause QT prolongation, torsade de pointes, heart failure, and rhabdomyolysis (18, 22, 23). In Brazil, fluconazole is the most commonly prescribed medication for the treatment of onychomycosis, however, its use is considered off-label in the United States of America (24, 25). Fluconazole inhibits the CYP3A4 and P2C9 (CYP2C9) systems of CYP450, thus interacting with other drugs that are metabolized through these pathways (26). The most frequently reported adverse reactions include nausea, vomiting, diarrhea, headache, and skin rash (27). The occurrence of hepatotoxicity, QT prolongation, and the Stevens-Johnson syndrome is sometimes reported (27, 28).

Topical antifungals rarely exhibit adverse effects and have negligible systemic absorption (13, 24, 29, 30). However, its use as monotherapy is limited to superficial white onychomycosis and less severe clinical manifestations, in which less than 80% of the nail plate is affected, in clinical forms in which there is no involvement of the lunula, or when systemic antifungals are contraindicated (18, 24). This is the most recommended presentation to treat older patients, children, pregnant women, and patients with chronic kidney disease undergoing renal replacement therapy, even though its effectiveness is associated with low rates of complete cure (18).

The most recommended topical antifungal agents for the treatment of onychomycosis are ciclopirox olamine and amorolfine in the form of lacquer and, to a lesser extent, tioconazole solution (13, 15, 24, 29, 31). Drugs such as efinaconazole, tavaborole, and luliconazole are alternative medicines in the form of solutions, but they are still not available in many countries, including Brazil (13, 15, 24, 29, 31). Nail debridement is considered an adjuvant form of topical therapy that aims to reduce the fungal mass and favor the penetration of medications into the deeper layers of the nail bed (32–34). Nail debridement is mainly indicated in the presence of dermatophytoma or nail plate hyperkeratosis (33, 35). Such procedure is carried out through several mechanisms, including: (a) cutting part of the nail that is detached from the nail bed; (b) mechanical abrasion of the nail plate using water sandpaper, and (c) chemical debridement applying urea-based formulations followed by occlusion (36, 37). Total nail avulsion is avoided due to the potential damage to the nail matrix, which can result in permanent dystrophy. In cases where nail debridement is necessary, the nail bed is unprotected and the esthetic problem remains, in addition, there is a loss of counterpressure in the nail bed, which can lead to increased lateral growth of the nail plate, thus the use of prosthetic nail is recommended (36, 37). Different types of therapy can be associated according to the needs of each case, aiming to improve the treatment effectiveness, especially in cases that are not responsive to monotherapy or with severe clinical phenotypes (38).

The main risk factors for the occurrence of onychomycosis include hyperhidrosis, use of public showers, use of closed shoes, micro trauma caused by footwear, tinea pedis, morphologically altered nails, psoriasis, peripheral vascular disease, obesity, and situations involving some immunological deficiency such as diabetes mellitus, advanced age, malignancies, chemotherapy, acquired human immunodeficiency syndrome, transplants, and hemodialysis (1, 3, 34, 39–45). Dialysis chronic kidney disease is associated with an immune deficiency related to uremia and increased oxidative stress that affects the innate and adaptive immune systems in ways similar to premature aging (46–48). The biological age of the T cell system of patients with chronic kidney disease on dialysis is approximately 20 years ahead of their chronological age, with a reduction in the production of naïve T cells, an increase in the number of memory T cells and changes in the compartment of regulatory T cells (49–51). In the same context, there is a reduction in the proliferative response to antigenic stimuli and the expression of cell surface markers that favor apoptosis (51). The patient develops a pro-inflammatory state with the increase and activation of innate immune system cells, such as monocytes and granulocytes, however, with compromised functionality (52). On the other hand, a reduction in the quantity and functionality of dendritic cells present in the skin and circulation is observed (53). Therefore, the decline in renal function decreases the effectiveness of the immune system and generates a pro-inflammatory milieu with notable consequences such as endothelial dysfunction, systemic inflammation, and susceptibility to infections, including fungal infections (3, 39, 41).

In view of the challenges encountered in the treatment of onychomycosis in patients with chronic kidney disease on dialysis, we carried out a phase II clinical trial that proposes cleaning and debridement of the affected hallux nail, followed by the placement of a nail prosthesis molded with acrylic reconstruction gel containing terbinafine at a 2% concentration, to obtain prolonged exposure of the tissues to the antifungal agent. Simultaneously, this technique aims to protect the debrided nail bed and improve the nail’s esthetic appearance.

## 2 Methods

### 2.1 Inclusion and exclusion criteria

Chronic renal dialysis patients undergoing treatment at the Hemodialysis Center of the São Francisco de Assis University Hospital in Bragança Paulista, São Paulo, Brazil were selected to participate in the study. The participants were over 18 years old and had hallux onychomycosis whose treatment involved debridement of the nail plate (dermatophytoma, subungual keratosis with a thickness of +2 mm or extensive onycholysis).

All patients at the Hemodialysis Center were initially evaluated through a clinical examination carried out by a medical professional. Those who presented nail morphological abnormalities suggestive of onychomycosis in the hallux underwent collection of nail fragments to perform a direct mycological examination and, subsequently, an additional sample was obtained to perform fungal culture to identify the etiological agent. None of the participants used antifungals to treat the onychomycosis close to the time of collection.

The exclusion criteria included clinical forms of onychomycosis that did not require debridement, namely white superficial onychomycosis, endonyx, and mild distal subungual onychomycosis. Patients with secondary onychomycosis, chronic mucocutaneous candidosis, individuals infected with the human immunodeficiency virus, and patients with psychiatric illness or reduced level of consciousness were also excluded. The criterion for discontinuation was the appearance of allergic or irritative contact dermatitis triggered by the material used in the intervention.

Participants were volunteers and signed an informed consent form before the start of the study. The study was approved by the São Francisco University research ethics committee (n° 59763022.8.0000.5514 – Opinion n° 064544/2022).

### 2.2 Study protocol

Patients presenting dermatophytoma, subungual hyperkeratosis with a thickness of +2 mm or extensive distal/lateral onycholysis underwent debridement of the affected nail plate by cutting it with sterile nail pliers or sanding with an electric nail file with a disposable water sandpaper tip and /or sterile scissors/pliers, to reduce the extent of the fungal mass to be treated (36, 37). Next, a small disposable brush with plastic bristles was used to eliminate keratin residue from the nail folds and, subsequently, the nail was cleaned using a 90% alcohol solution. Then, a layer of acrylic gel containing 2% terbinafine was administered and molded into the desired shape using a brush dipped in a mixture of acrylic monomers (Monomer) (Acrylic Liquid Honey Girl®, Lagoa Santa, Minas Gerais, Brazil) to reconstitute the nail plate that was debrided, spreading a thin layer of acrylic gel over the remaining nail plate. After molding the nail prosthesis, the gel was exposed to an ultraviolet light-emitting diode (UV/LED) source at 48 W for 2 min to solidify and polymerize the gel and the nail containing the nail prosthesis was cleaned with 90% alcohol again. Finally, the nail prosthesis was polished with an electric file to obtain a uniform relief. The process described in the study has not been previously published and is an adaptation of an esthetic procedure (use of acrylic gel nails) for the treatment of a disease (ungual onychomycosis).

Participants were re-evaluated every 2 weeks (~14 days), with a new cleaning and filing of the nail apparatus and a new application of the acrylic polymer containing 2% terbinafine. The process was carried out thoroughly to avoid damaging the healthy nail plate. In cases where the growth of a thickened nail plate was observed, with changes in color, or that was not adhered to the nail bed, the altered portion was again filed and/or trimmed before a new prosthetic nail was applied.

All participants were monitored and underwent intervention for 11 months until the final evaluation. The evolution was monitored through photographic records and measurement of healthy nail growth. At the end of the proposed treatment period, a direct mycological examination and fungal culture were performed, and the images were compared.

The terbinafine used in the study was supplied by the Alquimia compounding pharmacy of Bragança Paulista, São Paulo, Brazil and was mixed with acrylic gel at the Laboratory of Molecular Biology and Genetics of the São Francisco University, in São Paulo, Brazil to obtain a concentration of 2% of the drug in the polymer.

**Figure 1:**
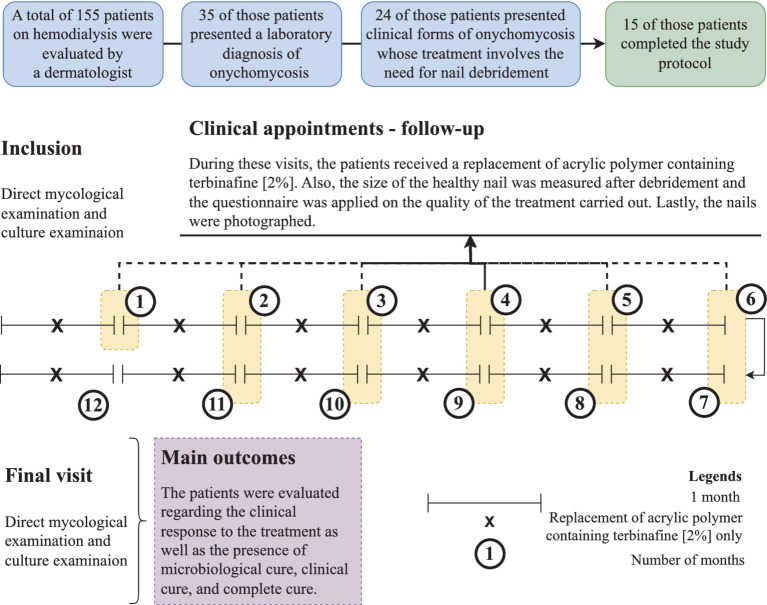
Study protocol showing the participants’ inclusion and their follow-up during the intervention period. Intervention was carried out with debridement of the nail followed by the application of acrylic polymer with 2% terbinafine. Participants were over 18 years old and presented onychomycosis of hallux, whose treatment involved debridement of the ungual plate (dermatophytoma, +2 mm thick subungual keratosis, or prolonged onycholysis). During the study period, nine participants discontinued the follow-up, as follows: **(a)** four participants died due to complications of the kidney disease, **(b)** three participants received a transplant and stopped to use the dialysis service, and **(c)** two participants decided not to continue in the study for personal reasons. Cure criteria were set based on the clinical progression and laboratory exam results, and were designed as follows: (clinical response) healthy nail 5 mm growth, (mycological cure) negative direct mycological and culture, (clinical cure) completely normal nail appearance, and (complete cure) presence of clinical and mycological cure.

The study’s complete protocol is shown in Figure 1.

### 2.3 Terbinafine availability assessment

Terbinafine at a concentration of 2% was added to polymerized acrylic gel and applied to healthy human nails. After 14 days, the nails were cut and sent for laboratory analysis to evaluate the bioavailability of the drug. The terbinafine was extracted from the nail by immersion in 100% acetonitrile, and subjected to an ultrasound bath for 10 min. The solvent was removed by nitrogen flow and the compound was resuspended in a solution containing 50% acetonitrile in water. The samples were analyzed by mass spectrometry from Integrated Pharmacology and Gastroenterology Unit — São Francisco University (triple quadrupole Xevo TQ-S, Waters Co., Milford, MA, United States) monitoring the terbinafine corresponding ion (292.4 m/z) and its fragment, after collision with argon gas, in positive mode, using a method previously validated by the research center to confirm the presence of the compound in the treated nail.

### 2.4 Onychomycosis patients’ demographic markers and clinical data

The study participants who were undergoing hemodialysis had data regarding age (years) and sex (male and female) collected. Furthermore, due to the presence of a positive result in the fungal culture exam, the following markers were also evaluated: (a) clinical type, (b) nail involvement severity score, and (c) measurement of the nail area.

The onychomycosis severity was assessed using the Onychomycosis Severity Index (OSI) system. The final score was obtained by multiplying the area of involvement score (range 0 to 5) by the proximity of the disease to the matrix score (range 1 to 5). Ten points were added for the presence of dermatophytoma, subungual hyperkeratosis with a thickness of +2 mm or a longitudinal stripe. After measuring the scores, the hemodialysis patients were classified into the following severity groups: (a) mild onychomycosis [score from 1 to 5], (b) moderate onychomycosis [score from 6 to 15], and (c) severe onychomycosis [score from 16 to 35] (54).

The nail plate area measurement was performed using the Image J, 1.47v image analysis software (NHI Bethesda, MD, United States). Measurements were taken immediately after the first debridement and repeated monthly and were used to evaluate the disease evolution. It seems relevant to observe that although debridement does not reduce the affected area, the elimination of subungual hyperkeratosis and dermatophytomas results in an immediate decrease in OSI.

### 2.5 Onychomycosis patients’ laboratory markers

Before starting the treatment, the nail containing morphological changes was cleaned with 70% alcohol. After the cleaning procedure, nail fragments and subungual debris were obtained by scraping the region of progression and confluence of healthy and affected tissue and of clinically altered areas, using an aluminum spatula and sterile nail scissors/pliers. The material obtained was deposited in sterile vials and sent to the clinical analysis laboratory.

For direct mycological examination, the material was bleached with potassium hydroxide to degrade the keratin and placed on a slide to visualize the fungal structures under an optical microscope. Analysis provided information on morphology, but species identification could not be determined by the examination. Fungal culture is considered the gold standard in onychomycosis diagnosis, as it allows the identification of the fungal species that affect the patient and shows the fungus viability. This examination employs Sabouraud and Sabouraud cycloheximide agar culture media. The culture is established between 20 and 25°C and the waiting time for growth might reach 4 weeks. The identification of the fungal agent is carried out through macromorphology and culture micromorphology analyses. The complete and detailed protocol was previously published (3).

Direct mycological examination and fungal culture were carried out at the São Francisco de Assis University Hospital clinical analysis laboratory in Bragança Paulista, São Paulo, Brazil, which is accredited by the Brazilian Health Surveillance Agency for this type of examination. In our data, patients on hemodialysis were considered to be affected by onychomycosis when direct mycological examination and fungal culture showed positive results.

### 2.6 Clinical response criteria (55)

Cure criteria were established based on clinical progression and laboratory tests and were outlined as follows: (clinical response) growth of 5 mm healthy nail, (mycological cure) negative direct mycological examination and fungal culture, (clinical cure) completely normal appearance of the nail and (complete cure) presence of clinical and mycological cure (55). To evaluate the efficacy of the treatment, none of the participants used antifungals to treat the onychomycosis close to the time of collection.

The participants’ perception of nail involvement was assessed using an open questionnaire with three questions. At the beginning of the treatment, the participants were asked about their foot care and nail hygiene habits. During the biweekly procedures, the participants were asked whether they felt pain or discomfort during the debridement and placement of nail prostheses, as well as in the subsequent days. Additionally, they were asked if they noticed any improvement in the appearance of their nails after starting treatment. The pain or discomfort during the debridement and placement of nail prostheses as well as the evidence of improvement in the appearance of their nails after starting treatment was described as *yes* or *no*.

## 3 Results

A total of 155 patients who were undergoing dialysis treatment during the study inclusion period were screened through clinical examination carried out by a medical doctor. Among the individuals, 64/155 (41.3%) patients presented nail changes that were indicative of onychomycosis of the halluces. Then, fragments of the affected nail were collected, and a direct mycological examination was performed with positive results in 35/64 (54.7%) of the individuals. All individuals with a positive direct mycological examination underwent a new collection of material for fungal culture and the etiological agent was identified. Out of this group, 24/35 (68.6%) individuals were indicated for debridement according to the inclusion criteria (Figure 1). During the study period, 9/24 (37.5%) participants discontinued the follow-up for the following reasons: (a) four participants died due to the kidney disease complications, (b) three participants were transplanted and stopped attending the dialysis service, and (c) two participants decided not to continue in the study for personal reasons. Thus, 15 individuals completed the study protocol.

In the study, 5/15 (33.3%) participants were female and 10/15 (66.6%) were male. The mean age ± SD (standard deviation) of the participants was 66.6 ± 12.3 years, ranging between 39 and 78 years. Initially, the clinical type of hallux involvement was assessed, with the presence of a distal subungual pattern and a mixed pattern observed in 9/15 (60.0%) and 6/15 (40.0%) study participants, respectively. All participants had a positive direct mycological test and only three participants had a negative culture test. Among the participants with a mixed pattern, the following mycological profile was evidenced at the time of inclusion: (i) *Candida* spp. (1 case), (ii) *T. rubrum* (2 cases), (iii) *T. mentagrophytes* (2 cases), and (iv) *Scytalidium* spp. (1 case). Regarding participants who presented a distal subungual pattern, the following mycological profile was observed at the time of inclusion: (i) *Candida* spp. (1 case), (ii) *T. rubrum* (1 case), (iii) *T. mentagrophytes* (2 cases), (iv) *Scytalidium* spp. (1 case), (v) *Penicillium marneffei* (1 case), and (vi) negative exam (3 cases). Demographic data and microbiological profile at the time of inclusion are summarized in Table 1.

**Table 1:** Description of the onychomycosis characteristic in chronic kidney patients on dialysis and outcomes observed in relation to the treatment of onychomycosis of hallux after debridement and application of acrylic polymer with 2% terbinafine.

Participant	Sex	Age (years)	Inclusion in the study			After the intervention – conclusion of the study					
			Fungal culture^a^	Clinical type	OSI (points)	OSI (points)	Direct mycological^a^	Fungal culture^b^	Clinical response^c^	Clinical cure^c^	Complete cure^c^
ABS	M	67	*Trichophyton rubrum*	Distal subungual	Severe (35)	Moderate (9)	Negative	*Trichophyton rubrum*	No	No	No
ACS	M	68	*Trichophyton rubrum*	Mixed pattern	Severe (35)	0	Negative	*Candida* spp.	Yes	Yes	No
DPZ	M	40	Negative	Distal subungual	Severe (26)	Moderate (16)	Negative	Negative	No	No	No
EFL	M	67	*Trichophyton rubrum*	Mixed pattern	Severe (19)	0	Negative	Negative	Yes	Yes	Yes
EAG	M	64	*Scytalidium* spp.	Mixed pattern	Severe (35)	Moderate (9)	Negative	*Penicillium manarffei*	No	No	No
ER	M	69	*Trichophyton mentagrophytes*	Distal subungual	Severe (35)	0	Negative	Negative	Yes	Yes	Yes
JPM	M	71	*Trichophyton mentagrophytes*	Distal subungual	Severe (19)	Moderate (6)	Negative	Negative	No	No	No
JAR	F	70	*Candida* spp.	Distal subungual	Severe (25)	Moderate (14)	Negative	Negative	No	No	No
LS	M	71	*Trichophyton mentagrophytes*	Mixed pattern	Severe (35)	Moderate (15)	Negative	*Candida* spp.	No	No	No
MR	F	47	Negative	Distal subungual	Severe (26)	Severe (19)	Negative	Negative	No	No	No
NB	M	78	Negative	Distal subungual	Moderate (14)	Moderate (6)	Negative	Negative	No	No	No
PB	M	45	*Trichophyton mentagrophytes*	Mixed pattern	Severe (35)	Moderate (12)	Negative	Negative	Yes	No	No
REA	F	62	*Scytalidium* spp.	Distal subungual	Severe (35)	Severe (35)	Yeast	Negative	No	No	No
SAN	F	45	*Candida* spp. non albicans	Mixed pattern	Severe (35)	Severe (25)	Negative	*Candida* spp.	No	No	No
VAC	F	47	*Penicillium marneffei*	Distal subungual	Severe (35)	Mild (4)*	Negative	Negative	Yes	Yes	Yes

M, male; F, female; ^a^all participants in this study presented positive direct mycological exam at the time of inclusion. The direct mycological and fungal culture exams were carried out at the São Francisco de Assis University Hospital clinical analysis laboratory in Bragança Paulista, São Paulo, Brazil, which is accredited by the Agência Vigilância Sanitária Brasileira (Brazilian Sanitation Surveillance Agency). ^b^the onychomycosis severity was assessed using the Onychomycosis Severity Index (OSI) system. The final score was obtained by multiplying the involvement area score (ranging from 0 to 5) by the disease proximity with the matrix score (ranging from 1 to 5). Ten points were added in the presence of subungual hyperkeratosis dermatophytoma + 2 mm thick or longitudinal band. After having the scores measured, the patients subjected to hemodialysis were classified in the following severity groups: mild onychomycosis [score from 1 to 5], moderate onychomycosis [score from 6 to 15], and severe onychomycosis [score from 16 to 35] (54). ^c^Cure criteria were set based on the clinical progression and laboratory exam results, and were designed as follows: (clinical response) healthy nail 5 mm growth, (mycological cure) negative direct mycological and culture, (clinical cure) completely normal nail appearance, and (complete cure) presence of clinical and mycological cure. *The patient presented less than 10% of the nail affected (clinical success).

At the time of inclusion, 14/15 (93.3%) participants had an OSI system classified as severe and only 1/15 (6.7%) participant had a score classified as moderate (Table 1). After the intervention, there was a change in the severity profile associated with the OSI system, with the participant initially classified as moderate OSI remaining in that classification and, among those classified as severe, 4/14 kept the OSI classification as severe, 6/14 started to be classified as moderate OSI, 1/14 were classified as mild OSI (with a degree of involvement below 10%) and 3/14 were classified as zero in the OSI system (clinical cure) (Table 1).

After the intervention period, the participants’ direct mycological examination result was negative in 14/15 (93.3%), and in one case there was the presence of yeast. The participant who presented the yeast had initially been diagnosed as infected with *Scytalidium* spp. Regarding fungal culture, after the intervention, participants with an initially negative result maintained the outcome profile. However, the remaining participants changed their microbiological profile, except in two cases in which the presence of *Candida* spp. and *T. rubrum* was observed in both moments evaluated. Furthermore, in 7/15 cases the culture became negative (1 case of *Candida* spp., 1 case of *Scytalidium* spp., 1 case of *P. marneffei*, 1 case of *T. rubrum*, and 3 cases of *T. mentagrophytes*), while in 3/15 cases there was a change in the etiological agent. The change occurred for participants who were initially classified as infected by *T. rubrum*, *Scytalidium* spp., and *T. mentagrophytes* and started to present *Candida* spp., *P. marneffei,* and *Candida* spp. in their fungal cultures, respectively. The result of the microbiological profile after the intervention is presented in Table 1.

**Figure 2:**
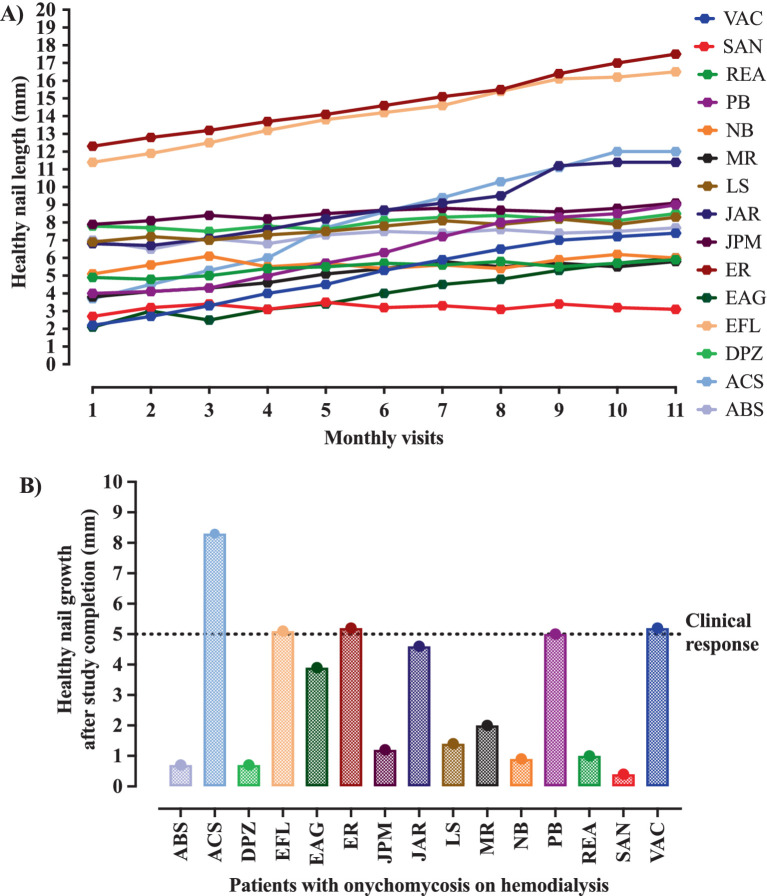
Evolution of the nail growth during the intervention period. The intervention was carried out with the nail debridement followed by the application of acrylic polymer with 2% terbinafine. The clinical response was positive in the presence of at least 5 mm healthy nail during the follow-up period. **(A)** All participants showed healthy nail growth. **(B)** The healthy nail growth was higher than that set as a positive response in only five cases, who showed 5-, 5.1-, 5.2-, 5.2-, and 8.3-mm nail growth, respectively, in the PB, EFL, ER, VAC, and ACS cases.

Clinical response was described in 5/15 (33.3%) participants; however, mycological cure, clinical cure, and complete cure were described, respectively for 9/15 (60.0%), 4/ 15 (26.7%) and 3/15 (20.0%) participants (Table 1). Regarding the clinical response, although all cases showed healthy nail growth (Figure 2A), the growth was higher than that recommended as a positive response (growth greater than 5 mm) in only five cases, among them, 5-, 5.1-, 5.2-, 5.2-, and 8.3-mm nail growth was observed, respectively, for PB, EFL, ER, VAC, and ACS cases (Figure 2B). Clinical response and cure rates were compared to values observed when using some systemic or topical drugs, as described in Table 2.

**Table 2:** Cure indices in the systemic and topic treatments of onychomycosis according to some of the drugs available in the market, and the treatment proposed in which debridement and application of acrylic polymer with 2% terbinafine was used in patients on hemodialysis due to the presence of chronic kidney disease*.

Systemic drug	Mycological cure	Clinical cure	Complete cure	Reference
Terbinafine	75.0%	81.3%	62.5%	(83)
Itraconazole	61.1%	77.8%	61.1%	(83)
Fluconazole	31.2%	37.5%	31.2%	(83)

Topical use drug	Mycological cure	Clinical cure	Complete cure	Reference
Amorolfine	51.8%	46.0%	44.6%	(98)
Ciclopirox	36.0%	Unavailable	12.0%	(98)
Efinaconazole	61.6%	56.6%	31.1%	(99)
Tavaborole	35.9%	Unavailable	Unavailable	(100)
Luliconazole	45.4%	32.8%	14.9%	(101)

Proposed treatment	Mycological cure	Clinical cure	Complete cure	Reference
Debridement followed by the application of acrylic polymer with 2% terbinafine	60.0%	26.7%	20.0%	ND

*The cure indices described in the studies are associated with the treatment, mainly of onychomycosis patients in the general population. However, in our study, only patients with moderate (one case) and severe (14 cases) onychomycosis phenotype and debridement indication for the disease management were included. ND, nothing to declare; %, percentage.

**Figure 3:**
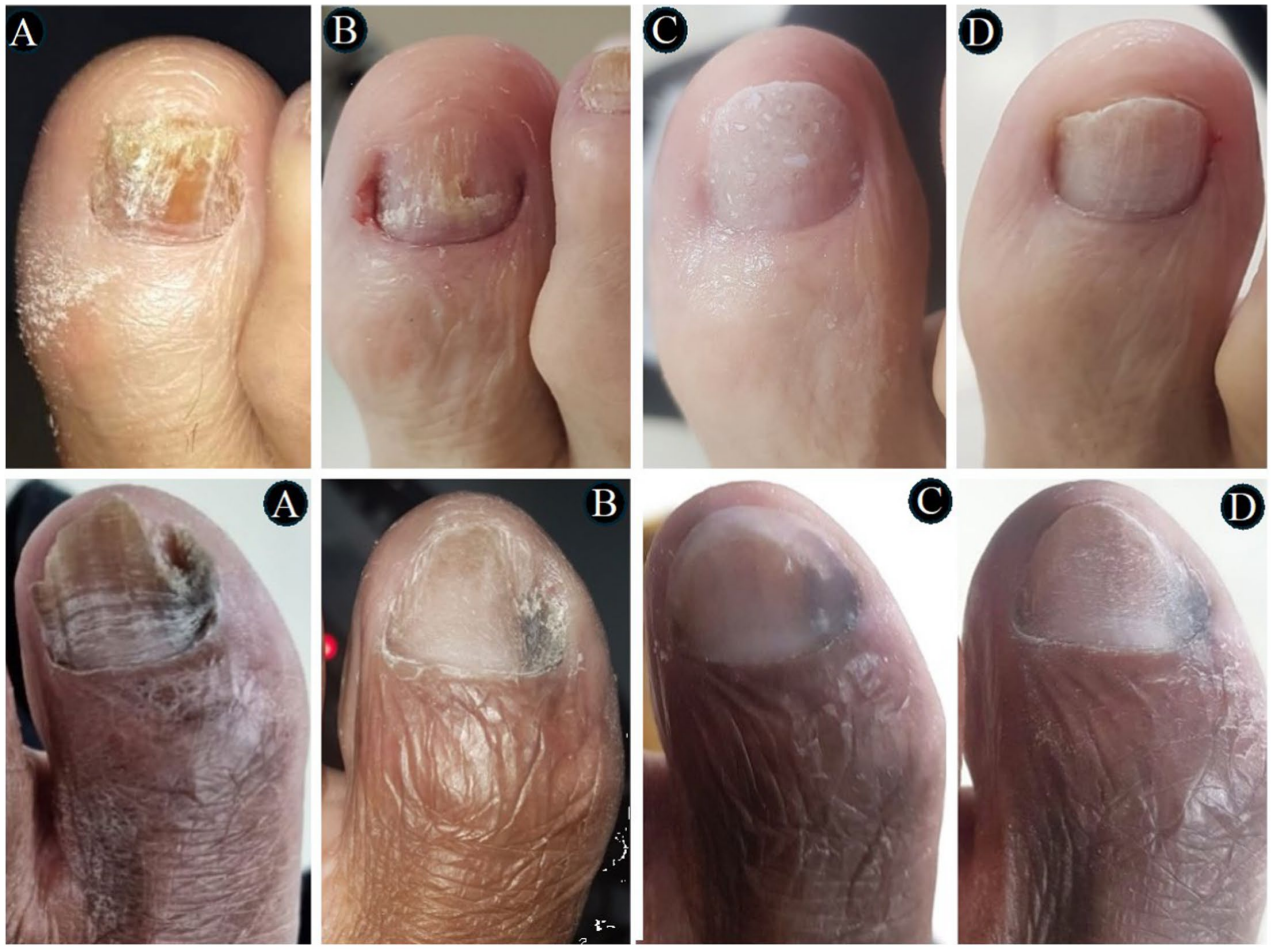
The images show two severe onychomycosis cases in which a complete cure was observed after the intervention period carried out with nail debridement followed by the application of acrylic polymer with 2% terbinafine. In both cases, we can see as follows: **(A)** the appearance of the hallux nail at the time of the initial evaluation – study inclusion period, when the direct mycological and fungal culture exams were carried out; **(B)** the appearance of the hallux nail after the first debridement and cleaning. The ungual plate debridement was carried out only in the cases indicated such as the presence of dermatophytoma, +2 mm thick subungual keratosis, or prolonged onycholysis; **(C)** the nail appearance during the intervention period. It seems relevant to observe that the polymerized acrylic gel with 2% terbinafine guaranteed the nail healthy appearance. **(D)** Nail appearance after 11 months of treatment with the polymerized acrylic gel with 2% terbinafine. Both cases resulted in a complete cure and absence of infectious agents according to the clinical and laboratory analyses.

**Figure 4:**
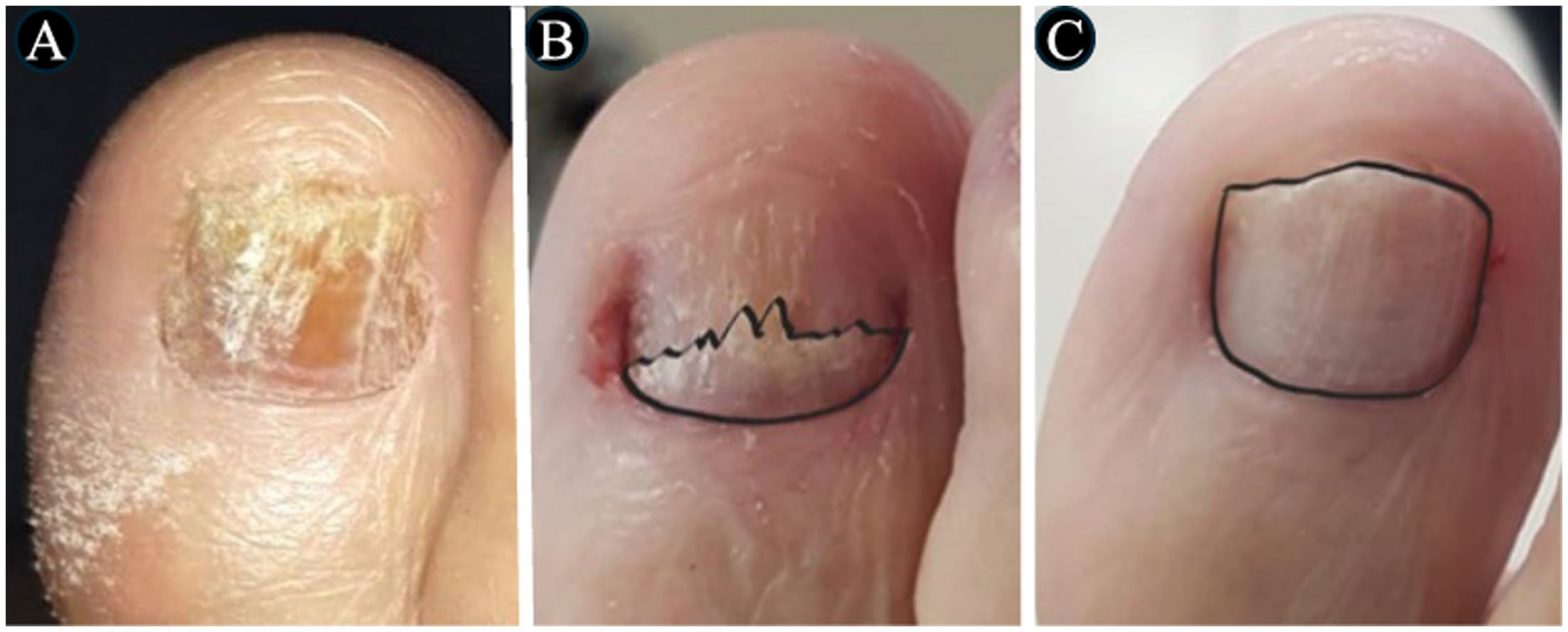
The images show a severe onychomycosis case in which a complete cure was observed after the intervention period carried out with nail debridement followed by the application of acrylic polymer with 2% terbinafine. **(A)** Appearance of the nail with onychomycosis before debridement in the presence of a 35-point Onychomycosis Severity Index (OSI). **(B)** Nail appearance immediately after the first debridement. The OSI value reduced from 35 (time of inclusion in the study) to 25 points, due to the removal of the subungual hyperkeratosis. Thus, to evaluate the therapeutical response, the remaining ungual plate area was measured aided by the Image J, 1,47v software (NHI Bethesda, MD, EUA). During the maintenance, whenever there was healthy nail growth, the increased area was added, but when dystrophic nail growth was observed, it was debrided again and, consequently the increased area removed was not recorded. **(C)** Nail appearance after the intervention period with a 0-point OSI. The onychomycosis severity was assessed using the OSI system. The final score was obtained by multiplying the involvement area score (ranging from 0 to 5) by the disease proximity with the matrix score (ranging from 1 to 5). Ten points were added in the presence of subungual hyperkeratosis dermatophytoma +2 mm thick or longitudinal band. After having the scores measured, the patients subjected to hemodialysis were classified in the following severity groups: mild onychomycosis [score from 1 to 5], moderate onychomycosis [score from 6 to 15], and severe onychomycosis [score from 16 to 35] (54).

**Figure 5:**
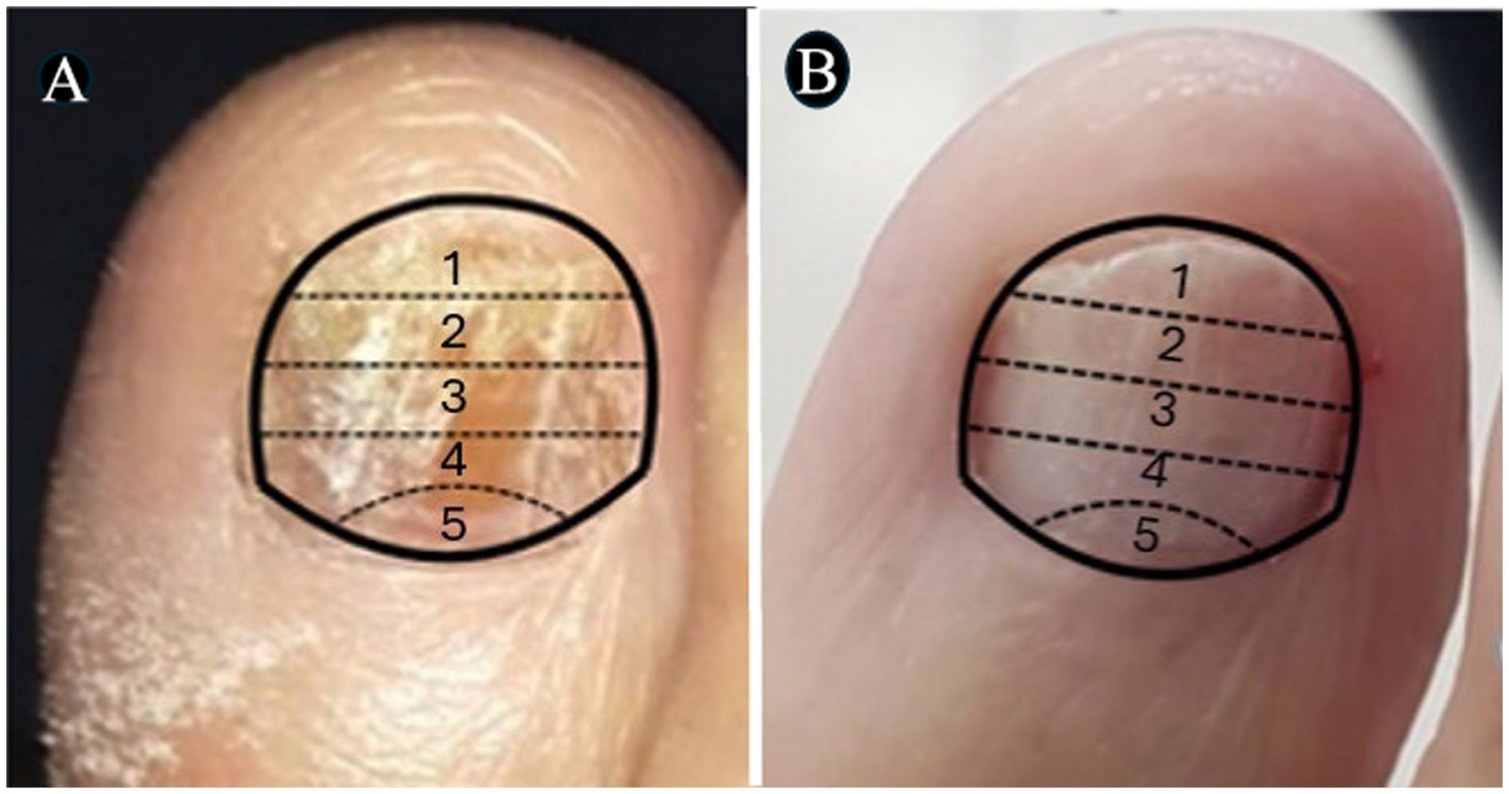
The images show a severe onychomycosis case in which a complete cure was observed after the intervention period carried out with nail debridement followed by the application of acrylic polymer with 2% terbinafine. **(A)** Nail appearance with mixed pattern onychomycosis, involvement of the ungual plate in the whole extension, in the presence of over 2 mm subungual hyperkeratosis. The Onychomycosis Severity Index (OSI) score was 35 points at the time of inclusion in the study. **(B)** Ungual plate appearance without clinical alteration after the intervention period with a 0-point OSI. The onychomycosis severity was assessed using the OSI. The final score was obtained by multiplying the involvement area score (ranging from 0 to 5) by the disease proximity with the matrix score (ranging from 1 to 5). Ten points were added in the presence of subungual hyperkeratosis dermatophytoma +2 mm thick or longitudinal band. After having the scores measured, the patients subjected to hemodialysis were classified in the following severity groups: mild onychomycosis [score from 1 to 5], moderate onychomycosis [score from 6 to 15], and severe onychomycosis [score from 16 to 35] (54).

**Figure 6:**
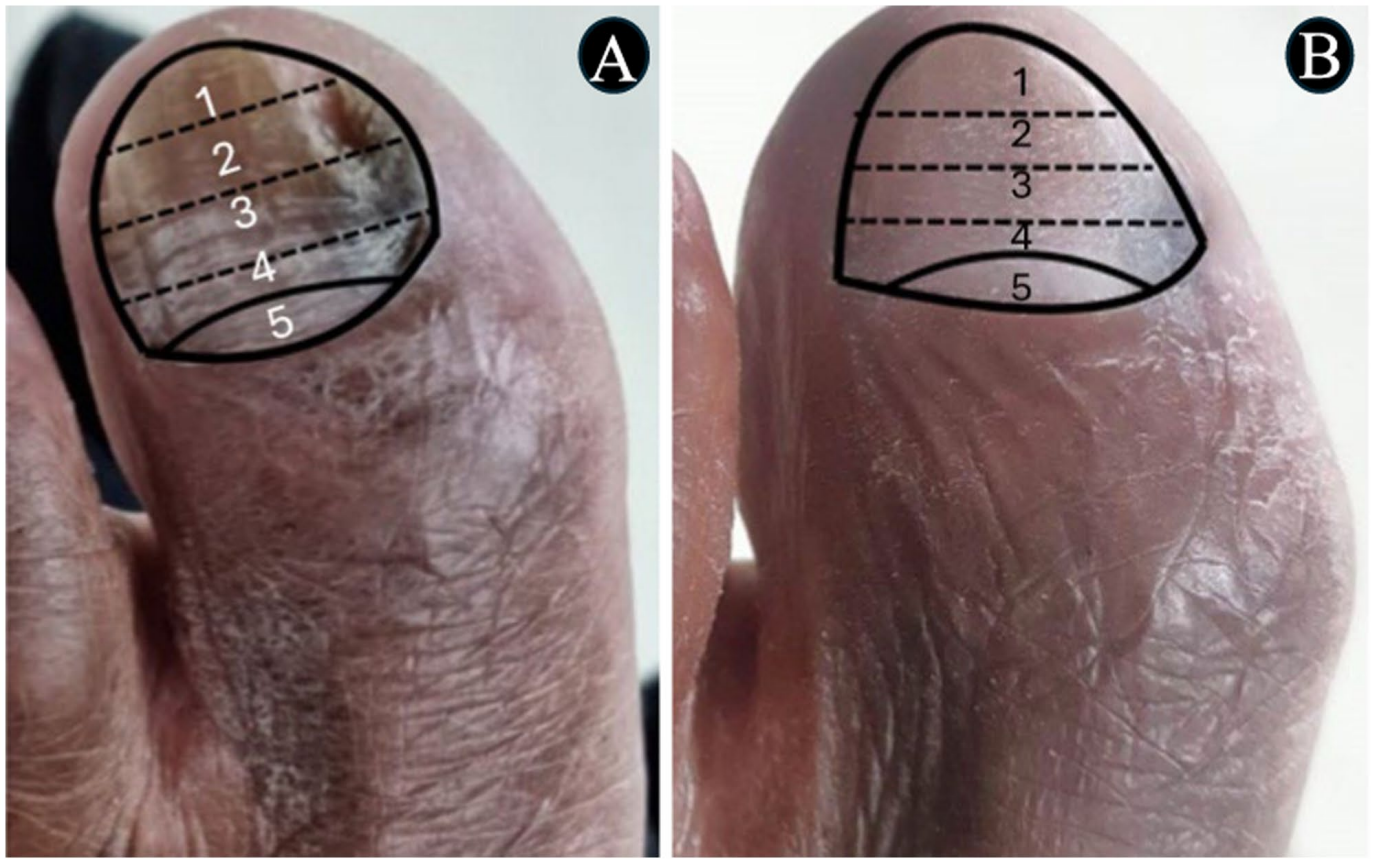
The images show a severe onychomycosis case in which a complete cure was observed after the intervention period carried out with nail debridement followed by the application of acrylic polymer with 2% terbinafine. **(A)** Mixed pattern onychomycosis with 26 to 50% involvement of the ungual plate, reaching segment 3. Presence of over 2 mm subungual hyperkeratosis. The Onychomycosis Severity Index (OSI) score was 19 points at the time of inclusion in the study. **(B)** After the intervention period, the ungual plate did not show clinical alterations and a 0-point OSI was recorded. The onychomycosis severity was assessed using the OSI system. The final score was obtained by multiplying the involvement area score (ranging from 0 to 5) by the disease proximity with the matrix score (ranging from 1 to 5). Ten points were added in the presence of subungual hyperkeratosis dermatophytoma +2 mm thick or longitudinal band. After having the scores measured, the patients subjected to hemodialysis were classified in the following severity groups: mild onychomycosis [score from 1 to 5], moderate onychomycosis [score from 6 to 15], and severe onychomycosis [score from 16 to 35] (54).

**Figure 7:**
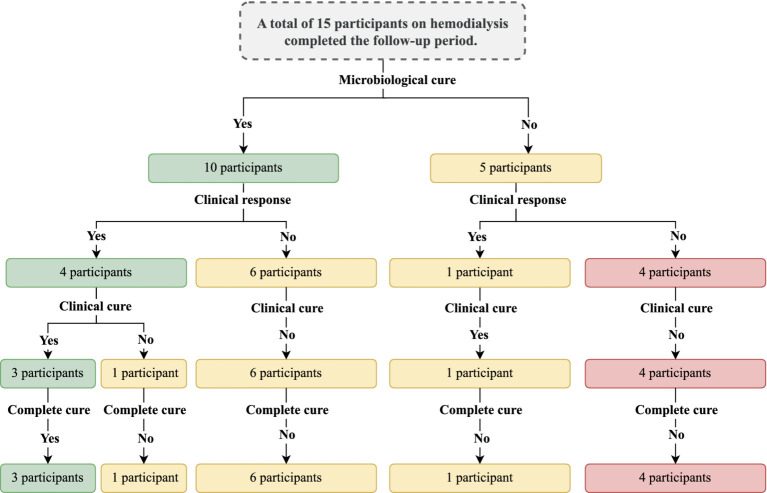
Summary of the main findings of the study with the outcomes observed. The outcomes were evaluated after the intervention period. The intervention was carried out with the nail debridement followed by the application of acrylic polymer with 2% terbinafine. Cure criteria were set based on the clinical progression and the laboratory exams and were designed as follows: (clinical response) healthy nail 5 mm growth, (mycological cure) negative direct mycological and fungal culture exams, (clinical cure) nail with a completely normal appearance, and (complete cure) presence of clinical and mycological cure.

In Figures 3–6, clinical cases are presented in which there was complete cure. In the images in Figure 3, the sequence shows the nail affected by onychomycosis, the nail after debridement, the nail containing the polymerized acrylic gel with 2% terbinafine, and, finally, complete healing showing the nail without signs of infectious agents through the clinical and laboratory analysis. Figures 4–6 presented the OSI score, the onychomycosis classification, and the clinical evolution. Figure 7 summarizes the main findings of the study.

**Figure 8:**
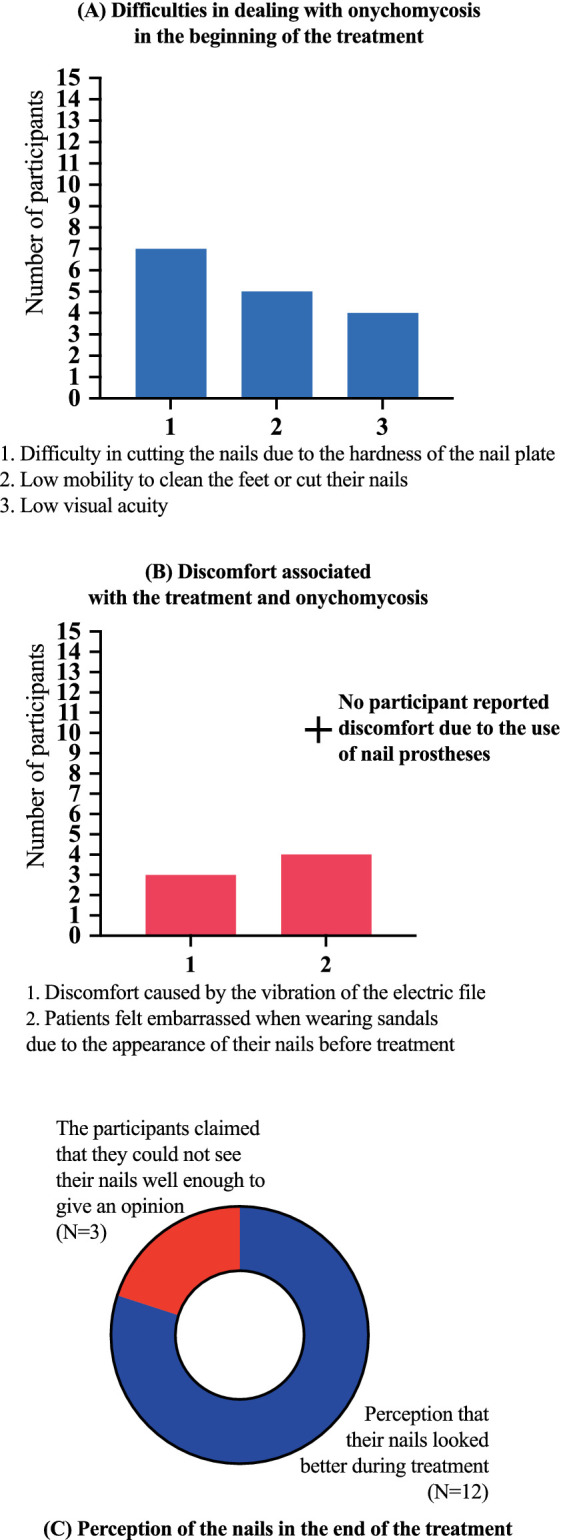
Results of the questionnaire about their perception of onychomycosis and the treatment development. **(A)** At the beginning of the treatment, 7/15 (46.7%) participants reported difficulty in cutting their nails due to the hardness of the nail plate, even with the help of family members, 5/15 (33.3%) reported that they did not have enough mobility to clean their feet or cut their nails, 4/15 (26.7%) reported that they did not see well enough and were therefore unable to clean their feet and cut their nails. **(B)** No participant reported discomfort due to the use of nail prostheses, 3/15 (20.0%) reported a feeling of discomfort caused by the vibration of the electric file, 4/15 (26.7%) reported that they felt embarrassed when wearing sandals due to the appearance of their nails before treatment. **(C)** A total of 12/15 (80.0%) participants reported the perception that their nails looked better during treatment, and 3/15 (20.0%) claimed that they could not see their nails well enough to give an opinion. No participant presented an allergic reaction or local irritation caused by the material used in the treatment. There were superficial and accidental ulcerations caused by electric sandpaper. No wound developed a secondary infection.

No participant presented an allergic reaction or local irritation caused by the material used in the treatment. There were superficial and accidental ulcerations caused by electric sandpaper. No wound developed a secondary infection. Participants responded to a questionnaire about their perception of onychomycosis and the treatment development. At the beginning of the treatment, 7/15 (46.7%) participants reported difficulty in cutting their nails due to the hardness of the nail plate, even with the help of family members, 5/15 (33.3%) reported that they did not have enough mobility to clean their feet or cut their nails, 4/15 (26.7%) reported that they did not see well enough and were therefore unable to clean their feet and cut their nails. No participant reported discomfort due to the use of nail prostheses, 3/15 (20.0%) reported a feeling of discomfort caused by the vibration of the electric file, 4/15 (26.7%) reported that they felt embarrassed when wearing sandals due to the appearance of their nails before treatment, 12/15 (80.0%) reported the perception that their nails looked better during treatment, and 3/15 (20.0%) claimed that they could not see their nails well enough to give an opinion (Figure 8).

**Figure 9:**
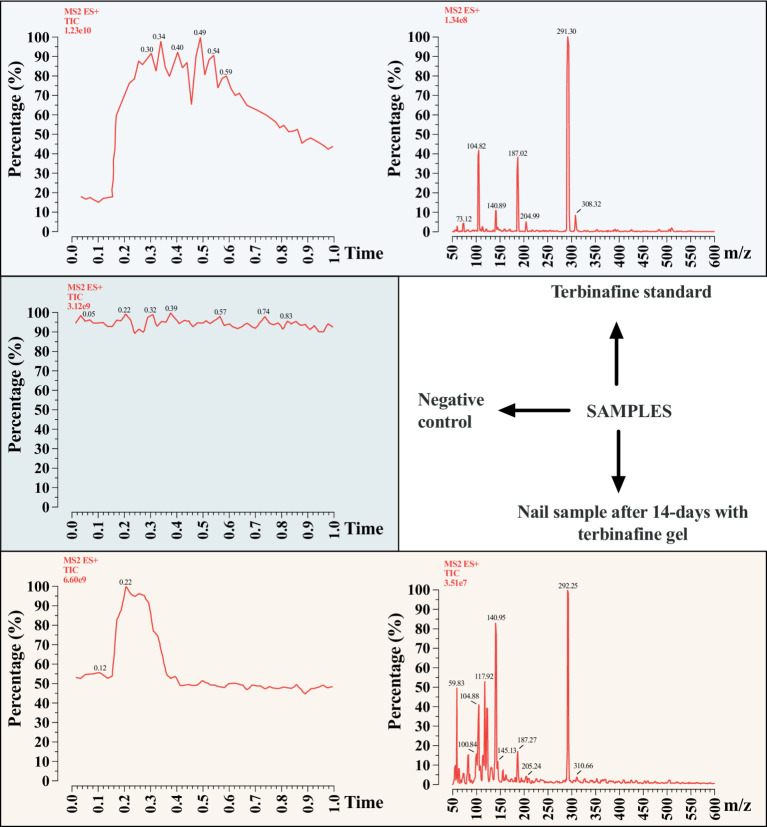
Identification of terbinafine in nail sample by mass spectrometry. Upper left = total ion current (TIC) of terbinafine standard. Upper right = MS2 profile of terbinafine, showing the parent ion (m/z 291) and its fragments (m/z 187). Middle left = TIC of negative control gel, showing no signal for terbinafine. Lower left = MS2 profile of nail sample containing terbinafine gel, showing the same ions as the standard.

We observed that in the gel in which terbinafine was inserted, the ion corresponding to the drug was detected, both in MS1 mode, with an increase in total ion current (TIC) in the retention time corresponding to the terbinafine standard, and in MS2 mode, in which the parental ion and its respective fragment were detected (291.30 m/z → 140.89 m/z). In the sample of a nail without the addition of terbinafine, used as a negative control, the presence of the ion was not detected in any of the modes, and in MS, even with the presence of a 292.18 m/z ion, its fragment was not visualized, therefore not corresponding to terbinafine. In a nail sample after 14 days with terbinafine gel, the 292.25 ion was detected, as well as its 141.40 m/z fragment, confirming the presence of the drug under these analysis conditions (Figure 9).

## 4 Discussion

In this study, most participants presented a mycological cure; however, clinical response, clinical cure, and, mainly, complete cure were infrequent. Furthermore, adverse effects associated with the treatment were not observed, and it was well tolerated by all individuals. The participants also reported that the treatment presented benefits in terms of self-esteem by promoting esthetic improvements associated with the onychomycosis presence.

In the literature, onychomycosis is described as a prevalent condition among individuals subjected to dialysis (3, 25, 41–43). The renal function decline reduces the immune system effectiveness and generates a pro-inflammatory milieu with the concomitant evolution to endothelial dysfunction, systemic inflammation, and susceptibility to infections, including fungal infections (3). In immunocompromised individuals, people with diabetes mellitus, as well as those with peripheral venous or arterial insufficiency, pedal onychomycosis assumes greater importance, as it can act as a gateway for bacterial agents, leading to greater complications (56). However, this condition may go unnoticed or be underreported as a health problem by these patients, whose attention is mainly focused on issues related to kidney disease and other serious comorbidities.

The onychomycosis management presents some particularities in chronic kidney disease patients on dialysis, as changes in immunity lead to an increased risk of becoming infected and developing more serious and difficult-to-treat forms of the disease, while the most effective treatments to date are carried out with medications for systemic use, which in the context of dialysis patients may require dose adjustments, present adverse effects and drug interactions (1, 5, 13, 18, 25, 29, 30, 34). Terbinafine is considered the most effective drug. It is metabolized by the liver, and 80% of the dose is excreted in urine and 20% in feces (16, 57–59). However, according to the literature, there are no extensive studies guiding safely the use of oral terbinafine in chronic renal patients on dialysis.

Itraconazole, the second drug with the highest cure rates, undergoes hepatic metabolism and is a strong CYP3A4 inhibitor (20, 21, 60, 61), with ~54% of the dose excreted in feces and 35% in urine (62). Itraconazole is not eliminated by intermittent dialysis, but rather by continuous hemofiltration (63). Fluconazole, another systemic use drug, is widely used in Brazil, undergoes glomerular filtration and tubular reabsorption, with 60 to 80% eliminated by the kidneys (27, 62). A single dose of fluconazole can be used in patients on dialysis without adjustments, but for prolonged use, dose adjustments are required since a 4-h hemodialysis session reduces plasma levels by ~40% (64).

Regarding the use of topical medications, considering the insignificant systemic absorption and the very low rate of adverse effects, they would represent the ideal therapy for chronic kidney disease patients on dialysis (4, 15). However, despite offering lower risk, they also present lower efficacy since they face the challenge of permeating the nail and reaching suitable concentrations in the nail bed (8, 65, 66). In the context of chronic renal patients on dialysis, there are also physical limitations such as visual impairment and mobility issues, which might result in difficulties in applying topical medication on toes.

Ointments and creams are easily removed in contact with water and are not suitable vehicles in the onychomycosis treatment. Due to their occlusive properties, varnish, enamel, adhesive, and films are able to increase nail hydration and keep the antifungal in the desired site for longer, favoring the medication permeation (10, 30, 65, 67). However, the cure indices using lacquer are still rather low (13, 30, 68, 69). Interestingly, these efficacy levels regard the general population when clinical phenotypes of diverse severity are considered. In such context, lower complete cure indices, such as those found in this study, must be evaluated considering the disease etiology, the patient’s age, the immunosuppression level, the probability of adverse effects, the possibility of using other medications, and, mainly, the cure rates resulting from conventional treatments in the population of kidney disease patients subjected to hemodialysis.

Several new strategies to promote nail permeation of drugs have been tested around the world. They are systems of a chemical nature, such as nanoparticles, microemulsions, hydrogels, and liposomes (70–73). Physical methods such as iontophoresis, laser, and ultrasound have also been tested (13, 74–77). However, no method was found that could tackle the problem in the short term (3, 5, 13, 25, 29), including from the esthetic standpoint. Some studies reported that harm to the quality of life associated with onychomycosis affects more women than men, and might in some cases generate embarrassment and social isolation (78–80).

Therefore, aiming to find an alternative for an immunocompromised population affected by severe forms of onychomycosis and with systemic treatment limitations, a new method was designed that combines nail plate debridement (required in these cases), with the application of a device that keeps contact between an antifungal agent and the nail bed for a long period. At the same time, this device also protects the debrided nail bed, thus improving the nail appearance. Subungual hyperkeratosis and prolonged onycholysis areas form a dead space where moist, keratin remains, and external residue tend to accumulate, resulting in an environment that favors fungus growth (81). When debridement is carried out, the fungal mass decreases and the environment is changed (82).

Terbinafine was selected for this study for being a broad-spectrum antifungal with greater efficacy against dermatophyte fungi, which are the most prevalent in the general population, as well as in the population selected for the intervention (16, 83). Higher terbinafine concentrations in the acrylic gel resulted in a more porous and fragile prosthesis consistency after polymerization, with consequent shorter fixation periods on the skin and nail plate, thus facilitating removal. On the other hand, the addition of 2% terbinafine at to the acrylic reconstruction gel resulted in a resilient nail prosthesis with satisfactory local fixation. We observed that even with a significantly reduced remaining nail plate after extensive debridement, a nail prosthesis could be attached. When applied, the acrylic gel adhered to the nail and nail bed, remaining that way for a variable period, depending on each patient’s characteristics and habits. Over time, the gel detaches, first from the nail bed, remaining attached only to the remaining nail plate. Consequently, a dead space is once again formed between the prosthesis and the nail bed, creating an environment similar to that observed during onycholysis, with the difference that the acrylic gel can be changed periodically and the area corresponding to the dead space can be sanitized, preventing the biofilm formation.

Nail hydration plays a significant role in its permeability. The literature describes that the flow of water in the fully hydrated nail plate is almost 6.5 times greater than in completely dry nails (84, 85). The gel contact with the ungual plate increases its hydration degree, making it more flexible (86, 87), and this effect favors the antifungal permeation. However, increased moist also promotes an environment that favors the development of fungi (88). In addition to hydrophilicity, molecular weight, ionization status, and the ability to bond to the keratin are also factors that affect the ability of the medication topically applied to permeate the nail (10, 30, 67, 89). Our study observed that, despite periodically repeating nail cleaning, most patients continued to present hyperkeratosis in the nail bed, an indicator of fungal activity in the epithelium, despite the terbinafine presence in the reconstruction gel.

The complete cure low efficacy as a result of the adopted treatment strategy can potentially be ascribed to factors such as the low local bioavailability of terbinafine, the absence of substances that facilitate the permeation of the active ingredient in the nail bed and nail plate, and the increase in local humidity, which creates an environment favorable to the growth of fungi. Host-related factors include immunodeficiency and limitations in maintaining adequate foot hygiene. It seems relevant to mention that achieving clinical cure is not always possible, particularly in cases of severe onychomycosis, previous damage to the nail matrix due to trauma resulting in permanent effects, secondary nail disease, and immunosuppression (2, 4, 8, 13).

In some participants of the study, there was minimal nail growth. Although lack of growth is not common, it can be associated with systemic health problems including the presence of liver dysfunction, kidney dysfunction, malnutrition, vitamin deficiencies, diabetes mellitus, autoimmune diseases, and hypothyroidism (90–93). In addition to these factors, factors such as age, genetics, and nutritional deficiencies may occur, as well as external and lifestyle factors, such as local trauma that causes damage to the nail matrix and contact and exposure with chemical products, such as excessive consumption of alcohol and tobacco or excessive heat that can affect nail growth (90–93). In our sample, patients had chronic kidney disease that is known to be associated with poor nail growth. Furthermore, unfortunately, we did not evaluate other conditions that could directly affect this growth and that could have affected the response to the protocol used in the study.

The occurrence of growth of a microorganism different from that initially isolated in the culture after treatment was observed in three patients. According to the literature, this fact can occur in mixed infections, with the culture medium favoring the growth of one of the etiological agents, in this case, dermatophytes (94–96). Thus, the agent identified in the first culture might be suppressed for being more sensitive to the antifungal used in the treatment, while in the post-treatment culture, a co-infecting etiological agent can be identified. The use of molecular methods to detect multiple agents is suggested as a solution (94, 97). However, in this study, only the direct mycological examination and the fungal culture were employed. Finally, one cannot completely rule out the possibility of contamination in the clinical analysis laboratory.

The study limitations include the fact that molecular methods were not used to identify the etiological agents. Furthermore, the number of participants was reduced, mainly due to the nature of the cases analyzed. The patients’ difficulty to keep suitable hygiene of their feet might have interfered with the evolution and outcomes observed. Despite the low cost of the materials and input used in the interventions, the protocol execution was quite hard and time consuming, which might make the procedure less accessible for routine treatment use. Moreover, the study population involves a complex phenotype to be considered regarding the onychomycosis management, namely, the severity of the kidney disease, commonly associated with countless comorbidities, age group (most patients in the study were older individuals), immunosuppression and genetic and environmental factors. It is important to note that resistance of biofilms in onychomycosis to commercially available antifungals has been documented in several studies, however, we did not evaluate the presence of biofilms. Also, alternative approaches such as photodynamic therapy, or the integration of nano systems might enhance treatment outcomes and should be implemented in future studies. Finally, we did not assess the susceptibility of the isolated fungi to terbinafine and other antifungals. These results could significantly enrich the study and provide stronger support for the discussion.

## 5 Conclusion

The addition of terbinafine to acrylic reconstruction gel for the manufacture of nail prostheses applied after debridement of moderate and severe forms of onychomycosis showed low efficacy as an isolated treatment in patients with chronic kidney disease on dialysis. On the other hand, most patients had a good perception of the appearance of their nails during treatment, even when it did not result in a therapeutic response or clinical cure. In future experiments, debridement and occlusion with the nail prosthesis may eventually be associated with formulations that combine antifungal medication with substances that facilitate nail and skin permeation that can be deposited between the prosthesis and the nail bed, and/or substances capable of reducing the local moist, making the nail bed a more hostile environment for fungal growth.

## Data Availability

The original contributions presented in the study are included in the article/supplementary material, further inquiries can be directed to the corresponding author/s.

## References

[1] Bodman MA Syed HA Krishnamurthy K. Onychomycosis. Treasure Island, FL: StatPearls Publishing (2024). 28722883

[2] Maskan Bermudez N Rodríguez-Tamez G Perez S Tosti A. Onychomycosis: old and new. J Fungi (Basel). (2023) 9:559. doi: 10.3390/jof9050559, PMID: 37233270 PMC10219498

[3] Bersano JMQB Cordeiro MG Marson FAL. Prevalence and risk predictors of onychomycosis in patients on hemodialysis: an observation, prospective, and unicenter study in Brazil. Front Med (Lausanne). (2023) 10:1268324. doi: 10.3389/fmed.2023.1268324, PMID: 38076229 PMC10701520

[4] Gupta AK Stec N Summerbell RC Shear NH Piguet V Tosti A. Onychomycosis: a review. J Eur Acad Dermatol Venereol. (2020) 34:1972–90. doi: 10.1111/jdv.16394 32239567

[5] Gupta AK Versteeg SG Shear NH. Onychomycosis in the 21st century: an update on diagnosis, epidemiology, and treatment. J Cutan Med Surg. (2017) 21:525–39. doi: 10.1177/1203475417716362, PMID: 28639462

[6] Lipner SR Scher RK. Onychomycosis. J Am Acad Dermatol. (2019) 80:835–51. doi: 10.1016/j.jaad.2018.03.062 29959961

[7] Lee DK Lipner SR. Optimal diagnosis and management of common nail disorders. Ann Med. (2022) 54:694–712. doi: 10.1080/07853890.2022.2044511, PMID: 35238267 PMC8896184

[8] Gupta AK Venkataraman M Talukder M. Onychomycosis in older adults: prevalence, diagnosis, and management. Drugs Aging. (2022) 39:191–8. doi: 10.1007/s40266-021-00917-8, PMID: 35102533

[9] Gupta AK Polla Ravi S Choi SY Konda A Cooper EA. Strategies for the enhancement of nail plate permeation of drugs to treat onychomycosis. J Eur Acad Dermatol Venereol. (2023) 37:243–55. doi: 10.1111/jdv.18638, PMID: 36196052

[10] Phan K Kaur K Wright K Tran S Stewart B Brown M. The penetrance of topical nail therapy: limitations and current enhancements. Clin Podiatr Med Surg. (2021) 38:535–40. doi: 10.1016/j.cpm.2021.06.004, PMID: 34538431

[11] Castillo C Masi MF Mishko A Sheltzer A Speer A Tran H. Biofilms and the nail unit. Clin Podiatr Med Surg. (2021) 38:529–33. doi: 10.1016/j.cpm.2021.06.005 34538430

[12] Gupta AK Foley KA. Evidence for biofilms in onychomycosis. G Ital Dermatol Venereol. (2019) 154:50–5. doi: 10.23736/S0392-0488.18.06001-7, PMID: 29683287

[13] Aggarwal R Targhotra M Kumar B Sahoo PK Chauhan MK. Treatment and management strategies of onychomycosis. J Mycol Med. (2020) 30:100949. doi: 10.1016/j.mycmed.2020.100949, PMID: 32234349

[14] Gupta AK Haas-Neill S Talukder M. The safety of oral antifungals for the treatment of onychomycosis. Expert Opin Drug Saf. (2023) 22:1169–78. doi: 10.1080/14740338.2023.2280137, PMID: 37925672

[15] Leung AKC Lam JM Leong KF Hon KL Barankin B Leung AAM. Onychomycosis: an updated review. Recent Patents Inflamm Allergy Drug Discov. (2020) 14:32–45. doi: 10.2174/1872213X13666191026090713, PMID: 31738146 PMC7509699

[16] Maxfield L Preuss CV Bermudez R. Terbinafine. Treasure Island, FL: StatPearls Publishing (2024). 31424802

[17] Abdelrahman S Gotschall R Kauffman R Leeder J Kearns G. Investigation of terbinafine as a CYP2D6 inhibitor in vivo. Clin Pharmacol Ther. (1999) 65:465–72. doi: 10.1016/S0009-9236(99)70065-2, PMID: 10340911

[18] Ricardo JW Lipner SR. Safety of current therapies for onychomycosis. Expert Opin Drug Saf. (2020) 19:1395–408. doi: 10.1080/14740338.2020.1829592, PMID: 32990062

[19] Etgü F. Retrospective analysis of liver enzyme abnormalities in patients prescribed Terbinafine and Itraconazole for onychomycosis. Cureus. (2023) 15:e44914. doi: 10.7759/cureus.44914, PMID: 37818526 PMC10561530

[20] Wang Y Lipner SR. Retrospective analysis of adverse events with systemic onychomycosis medications reported to the United States Food and Drug Administration. J Dermatolog Treat. (2021) 32:783–7. doi: 10.1080/09546634.2019.1708242 31865826

[21] Xie S-L Zhu X Gao N Lin Q Chen C Yang Y-J. Genetic variations of CYP3A4 on the metabolism of itraconazole in vitro. Food Chem Toxicol. (2023) 181:114101. doi: 10.1016/j.fct.2023.114101, PMID: 37863381

[22] Ezad S Cheema H Collins N. Statin-induced rhabdomyolysis: a complication of a commonly overlooked drug interaction. Oxf med case reports. 2018, omx104 (2018) 2018. doi: 10.1093/omcr/omx104, PMID: 29593874 PMC5853001

[23] Salem M Reichlin T Fasel D Leuppi-Taegtmeyer A. Torsade de pointes and systemic azole antifungal agents: analysis of global spontaneous safety reports. Glob Cardiol Sci Pract. (2017) 2017:11. doi: 10.21542/gcsp.2017.11, PMID: 29644223 PMC5871400

[24] Falotico JM Lipner SR. Updated perspectives on the diagnosis and Management of Onychomycosis. Clin Cosmet Investig Dermatol. (2022) 15:1933–57. doi: 10.2147/CCID.S362635, PMID: 36133401 PMC9484770

[25] Lipner SR Scher RK. Onychomycosis. J Am Acad Dermatol. (2019) 80:853–67. doi: 10.1016/j.jaad.2018.05.1260 29959962

[26] Niwa T Imagawa Y Yamazaki H. Drug interactions between nine antifungal agents and drugs metabolized by human cytochromes P450. Curr Drug Metab. (2014) 15:651–79. doi: 10.2174/1389200215666141125121511, PMID: 25429674

[27] Govindarajan A Bistas KG Ingold CJ Aboeed A. Fluconazole. Treasure Island, FL: StatPearls Publishing (2024). 30725843

[28] Godfrey H Jedlowski P Thiede R. Medication associations with severe cutaneous adverse reactions: a case/non-case analysis using the FDA adverse event reporting system. J Cutan Med Surg. (2024) 28:51–8. doi: 10.1177/12034754231220931, PMID: 38189282

[29] Beuscher TL Kelechi TJ. Onychomycosis. J Wound Ostomy Continence Nurs. (2019) 46:333–5. doi: 10.1097/WON.0000000000000556 31274864

[30] Costa P. de Mendes V. Veiga F. F. Negri M. Svidzinski T. I. E. (2022). Relevant insights into onychomycosis’ pathogenesis related to the effectiveness topical treatment. Microb Pathog 169,:105640. doi: 10.1016/j.micpath.2022.105640, PMID: 35716926

[31] Geizhals S Lipner SR. A timeline of onychomycosis therapy and future directions. Skinmed. (2020) 18:367–71. PMID: 33397567

[32] Frazier WT Santiago-Delgado ZM Stupka KC. Onychomycosis: rapid evidence review. Am Fam Physician. (2021) 104:359–67. PMID: 34652111

[33] Gupta AK Wang T Cooper EA. Dermatophytomas: clinical overview and treatment. J Fungi (Basel). (2022) 8:742. doi: 10.3390/jof8070742, PMID: 35887497 PMC9323405

[34] Zaraa I Dehavay F Richert B. Onychomycosis. Hand Surg Rehabil. (2024) 43:101638. doi: 10.1016/j.hansur.2024.101638 38218374

[35] Hm A. Excellent response of Dermatophytoma and nail splitting to nail plate debridement plus topical Ciclopirox Olamine 1% solution. Skin Appendage Disord. (2021) 7:127–30. doi: 10.1159/000512037, PMID: 33796559 PMC7991492

[36] Baran R Hay RJ Garduno JI. Review of antifungal therapy and the severity index for assessing onychomycosis: part I. J Dermatolog Treat. (2008) 19:72–81. doi: 10.1080/09546630701243418, PMID: 18484426

[37] Lin K Lipner SR. Painless technique for debridement of onychodystrophic nails. J Am Acad Dermatol. (2020) 82:e39–40. doi: 10.1016/j.jaad.2019.05.069, PMID: 31150717

[38] Falotico JM Lapides R Lipner SR. Combination therapy should be reserved as second-line treatment of onychomycosis: a systematic review of onychomycosis clinical trials. J Fungi (Basel). (2022) 8:279. doi: 10.3390/jof8030279, PMID: 35330281 PMC8949799

[39] Anees M Butt G Gull S Nazeer A Hussain I Ibrahim M. Factors affecting dermatological manifestations in patients with end stage renal disease. J Coll Physicians Surg Pak. (2018) 28:98–102. doi: 10.29271/jcpsp.2018.02.98, PMID: 29394966

[40] Bunyaratavej S Srinonprasert V Kiratiwongwan R Wongdama S Leeyaphan C. Onychomycosis in older adults: the age and associated factors affecting the complete cure rate. Australas J Dermatol. (2022) 63:74–80. doi: 10.1111/ajd.13686, PMID: 34398469

[41] Eftekhari H Haghdar Saheli Y Ashoobi MT Mahjoob M Kazemnezhad Leyli E Bagheri Toolaroud P. The prevalence of onychomycosis in patients with chronic renal failure undergoing dialysis: a cross-sectional study. Heliyon. (2024) 10:e25737. doi: 10.1016/j.heliyon.2024.e25737, PMID: 38434055 PMC10906168

[42] Filho AMS Ventura CG Criado PR Del Negro GB Freitas RS Luiz OC. Hemodialysis and kidney transplantation as predisposing conditions to onychomycosis. Nephron. (2017) 137:38–46. doi: 10.1159/000475674, PMID: 28609764

[43] Kuvandik G Çetin M Genctoy G Horoz M Duru M Akcali C. The prevalance, epidemiology and risk factors for onychomycosis in hemodialysis patients. BMC Infect Dis. (2007) 7:102. doi: 10.1186/1471-2334-7-102, PMID: 17760994 PMC2206043

[44] Navarro-Pérez D Tardáguila-García A García-Oreja S López-Moral M García-Madrid M Lázaro-Martínez JL. Onychomycosis associated with diabetic foot syndrome: a systematic review. Mycoses. (2023) 66:459–66. doi: 10.1111/myc.13577, PMID: 36790078

[45] Ogawa T Matsuda A Ogawa Y Tanaka R. Risk factors for the development of tinea pedis and onychomycosis: real-world evidence from a single-podiatry center, large-scale database in Japan. J Dermatol. (2024) 51:30–9. doi: 10.1111/1346-8138.16991, PMID: 37904622 PMC11483953

[46] Betjes MGH. Immune cell dysfunction and inflammation in end-stage renal disease. Nat Rev Nephrol. (2013) 9:255–65. doi: 10.1038/nrneph.2013.44 23507826

[47] Kong L Andrikopoulos S MacIsaac RJ Mackay LK Nikolic-Paterson DJ Torkamani N. Role of the adaptive immune system in diabetic kidney disease. J Diabetes Investig. (2022) 13:213–26. doi: 10.1111/jdi.13725, PMID: 34845863 PMC8847140

[48] Zoccali C Mallamaci F. Innate immunity system in patients with cardiovascular and kidney disease. Circ Res. (2023) 132:915–32. doi: 10.1161/CIRCRESAHA.122.321749, PMID: 37053283

[49] Sampani E Vagiotas L Daikidou D-V Nikolaidou V Xochelli A Kasimatis E. End stage renal disease has an early and continuous detrimental effect on regulatory T cells. Nephrology (Carlton). (2022) 27:281–7. doi: 10.1111/nep.13996, PMID: 34781412

[50] Yang TO Chuang Y-F Chiu Y-L. T-cell aging in end-stage renal disease: an evolving story with CMV. Med Microbiol Immunol. (2019) 208:281–7. doi: 10.1007/s00430-019-00596-8, PMID: 30903371

[51] Zakaria MM Derbala SA Salem AE El-Agroudy AE El-Tantawy FM. Inflammatory markers in chronic kidney disease and end stage renal disease patients. Mol Biol Rep. (2021) 48:6857–62. doi: 10.1007/s11033-021-06684-4 34472006

[52] Gusev E Solomatina L Zhuravleva Y Sarapultsev A. The pathogenesis of end-stage renal disease from the standpoint of the theory of general pathological processes of inflammation. Int J Mol Sci. (2021) 22:11453. doi: 10.3390/ijms222111453, PMID: 34768884 PMC8584056

[53] Kim JU Kim M Kim S Nguyen TT Kim E Lee S. Dendritic cell dysfunction in patients with end-stage renal disease. Immune Netw. (2017) 17:152–62. doi: 10.4110/in.2017.17.3.152, PMID: 28680376 PMC5484645

[54] Carney C Tosti A Daniel R Scher R Rich P DeCoster J. A new classification system for grading the severity of onychomycosis: onychomycosis severity index. Arch Dermatol. (2011) 147:1277–82. doi: 10.1001/archdermatol.2011.267, PMID: 22106113

[55] Gupta AK Studholme C. How do we measure efficacy of therapy in onychomycosis: patient, physician, and regulatory perspectives. J Dermatolog Treat. (2016) 27:498–504. doi: 10.3109/09546634.2016.1161156, PMID: 27043266

[56] Matricciani L Talbot K Jones S. Safety and efficacy of tinea pedis and onychomycosis treatment in people with diabetes: a systematic review. J Foot Ankle Res. (2011) 4:26. doi: 10.1186/1757-1146-4-26, PMID: 22136082 PMC3248359

[57] Gupta AK Polla Ravi S Talukder M Mann A. Effectiveness and safety of oral terbinafine for dermatophyte distal subungual onychomycosis. Expert Opin Pharmacother. (2024) 25:15–23. doi: 10.1080/14656566.2024.2305304, PMID: 38221907

[58] Humbert H Cabiac MD Denouël J Kirkesseli S. Pharmacokinetics of terbinafine and of its five main metabolites in plasma and urine, following a single oral dose in healthy subjects. Biopharm Drug Dispos. (1995) 16:685–94. doi: 10.1002/bdd.2510160807, PMID: 8573687

[59] Kovarik JM Mueller EA Zehender H Denouël J Caplain H Millerioux L. Multiple-dose pharmacokinetics and distribution in tissue of terbinafine and metabolites. Antimicrob Agents Chemother. (1995) 39:2738–41. doi: 10.1128/AAC.39.12.2738, PMID: 8593011 PMC163021

[60] Gupta AK Foley KA Mays RR Shear NH Piguet V. Monotherapy for toenail onychomycosis: a systematic review and network meta-analysis. Br J Dermatol. (2020) 182:287–99. doi: 10.1111/bjd.18155, PMID: 31120134

[61] Piérard GE Arrese JE Piérard-Franchimont C. Itraconazole. Expert Opin Pharmacother. (2000) 1:287–304. doi: 10.1517/14656566.1.2.287 11249550

[62] Bellmann R Smuszkiewicz P. Pharmacokinetics of antifungal drugs: practical implications for optimized treatment of patients. Infection. (2017) 45:737–79. doi: 10.1007/s15010-017-1042-z, PMID: 28702763 PMC5696449

[63] Willems L van der Geest R de Beule K. Itraconazole oral solution and intravenous formulations: a review of pharmacokinetics and pharmacodynamics. J Clin Pharm Ther. (2001) 26:159–69. doi: 10.1046/j.1365-2710.2001.00338.x 11422598

[64] Oono S Tabei K Tetsuka T Asano Y. The pharmacokinetics of fluconazole during haemodialysis in uraemic patients. Eur J Clin Pharmacol. (1992) 42:667–9. doi: 10.1007/BF00265934, PMID: 1623910

[65] Issa NT Kircik L. Individual article: updated review of topical pharmaceuticals and complementary and alternative medications for the treatment of onychomycosis in both general and special populations in the United States. J Drugs Dermatol. (2023) 22:SF378719-SF378719s10. doi: 10.36849/JDD.8719, PMID: 37683068

[66] Thomas J Peterson GM Christenson JK Kosari S Baby KE. Antifungal drug use for onychomycosis. Am J Ther. (2019) 26:e388–96. doi: 10.1097/MJT.0000000000000696 31082864

[67] Murdan S. Drug delivery to the nail following topical application. Int J Pharm. (2002) 236:1–26. doi: 10.1016/s0378-5173(01)00989-9, PMID: 11891066

[68] Gupta AK Mays RR Versteeg SG Shear NH Piguet V. Update on current approaches to diagnosis and treatment of onychomycosis. Expert Rev Anti-Infect Ther. (2018) 16:929–38. doi: 10.1080/14787210.2018.1544891, PMID: 30411650

[69] Lipner SR Joseph WS Vlahovic TC Scher RK Rich P Ghannoum M. Therapeutic recommendations for the treatment of toenail onychomycosis in the US. J Drugs Dermatol. (2021) 20:1076–84. doi: 10.36849/JDD.6291, PMID: 34636509

[70] Barot BS Parejiya PB Patel HK Mehta DM Shelat PK. Microemulsion-based antifungal gel delivery to nail for the treatment of onychomycosis: formulation, optimization, and efficacy studies. Drug Deliv Transl Res. (2012) 2:463–76. doi: 10.1007/s13346-012-0109-8, PMID: 25787325

[71] Kesharwani P Fatima M Singh V Sheikh A Almalki WH Gajbhiye V. Itraconazole and Difluorinated-curcumin containing chitosan nanoparticle loaded hydrogel for amelioration of onychomycosis. Biomimetics (Basel). (2022) 7:206. doi: 10.3390/biomimetics7040206, PMID: 36412734 PMC9680304

[72] Tuncay Tanrıverdi S Hilmioğlu Polat S Yeşim Metin D Kandiloğlu G Özer Ö. Terbinafine hydrochloride loaded liposome film formulation for treatment of onychomycosis:in vitroandin vivoevaluation. J Liposome Res. (2016) 26:163–73. doi: 10.3109/08982104.2015.1067892 26226352

[73] Valdez AF Zamith-Miranda D Nimrichter L Nosanchuk JD. Micro- and nanoparticles as platforms for the treatment of fungal infections: present and future perspectives. Future Microbiol. (2023) 18:1007–11. doi: 10.2217/fmb-2023-0079, PMID: 37721209 PMC10718170

[74] Dhamoon RK Popli H Gupta M. Novel drug delivery strategies for the treatment of onychomycosis. Pharm Nanotechnol. (2019) 7:24–38. doi: 10.2174/2211738507666190228104031, PMID: 31092174 PMC6691844

[75] Kline-Schoder A Le Z Zderic V. Ultrasound-enhanced Ciclopirox delivery for treatment of onychomycosis. Annu Int Conf IEEE Eng Med Biol Soc. (2018) 2018:5717–20. doi: 10.1109/EMBC.2018.8513552, PMID: 30441634

[76] Martins Andrade JF da Cunha Miranda T Cunha-Filho M Taveira SF Gelfuso GM Gratieri T. Iontophoresis application for drug delivery in high resistivity membranes: nails and teeth. Drug Deliv Transl Res. (2023) 13:1272–87. doi: 10.1007/s13346-022-01244-0 36209312

[77] Zhang J Lin P Li J Guo C Zhai J Zhang Y. Efficacy of laser therapy combined with topical antifungal agents for onychomycosis: a systematic review and meta-analysis of randomised controlled trials. Lasers Med Sci. (2022) 37:2557–69. doi: 10.1007/s10103-022-03561-9, PMID: 35484440

[78] Debbagh F Babokh F Sbai M El Mezouari EM Moutaj R. Impact of onychomycosis on the quality of life of patients. Curr Med Mycol. (2023) 9:39–44. doi: 10.18502/cmm.2023.345062.1430, PMID: 38375523 PMC10874484

[79] Lipner SR Falotico JM Konda A. On the basis of sex: impact and treatment of toenail onychomycosis in female patients. J Clin Aesthet Dermatol. (2023) 16:52–7. PMID: 37915332 PMC10617899

[80] Stewart CR Algu L Kamran R Leveille CF Abid K Rae C. Effect of onychomycosis and treatment on patient-reported quality-of-life outcomes: a systematic review. J Am Acad Dermatol. (2021) 85:1227–39. doi: 10.1016/j.jaad.2020.05.143 32502586

[81] Nagar R Nayak CS Deshpande S Gadkari RP Shastri J. Subungual hyperkeratosis nail biopsy: a better diagnostic tool for onychomycosis. Indian J Dermatol Venereol Leprol. (2012) 78:620–4. doi: 10.4103/0378-6323.100579, PMID: 22960819

[82] Grover C Bansal S Nanda S Reddy BSN Kumar V. Combination of surgical avulsion and topical therapy for single nail onychomycosis: a randomized controlled trial. Br J Dermatol. (2007) 157:364–8. doi: 10.1111/j.1365-2133.2007.08014.x 17573877

[83] Arca E Taştan HB Akar A Kurumlu Z Gür AR. An open, randomized, comparative study of oral fluconazole, itraconazole and terbinafine therapy in onychomycosis. J Dermatolog Treat. (2002) 13:3–9. doi: 10.1080/09546630252775171 12006131

[84] Walters KA Abdalghafor HM Lane ME. The human nail--barrier characterisation and permeation enhancement. Int J Pharm. (2012) 435:10–21. doi: 10.1016/j.ijpharm.2012.04.024, PMID: 22521879

[85] Walters KA Flynn GL Marvel JR. Physicochemical characterization of the human nail: permeation pattern for water and the homologous alcohols and differences with respect to the stratum corneum. J Pharm Pharmacol. (1983) 35:28–33. doi: 10.1111/j.2042-7158.1983.tb04258.x, PMID: 6131961

[86] Bhuptani RS Deshpande KM Patravale VB. Transungual permeation: current insights. Drug Deliv Transl Res. (2015) 6:426–39. doi: 10.1007/s13346-015-0259-6, PMID: 26419676

[87] Shivakumar HN Juluri A Desai BG Murthy SN. Ungual and transungual drug delivery. Drug Dev Ind Pharm. (2012) 38:901–11. doi: 10.3109/03639045.2011.637931, PMID: 22149347

[88] Zaias N Escovar SX Zaiac MN. Finger and toenail onycholysis. J Eur Acad Dermatol Venereol. (2015) 29:848–53. doi: 10.1111/jdv.12862, PMID: 25512134

[89] Chouhan P Saini TR. Hydroxypropyl- β -cyclodextrin: a novel Transungual permeation enhancer for development of topical drug delivery system for onychomycosis. J Drug Deliv. (2014) 2014:950358. doi: 10.1155/2014/950358, PMID: 25177500 PMC4142161

[90] Yousif J Farshchian M Potts GA. Oral nail growth supplements: a comprehensive review. Int J Dermatol. (2022) 61:916–22. doi: 10.1111/ijd.15807, PMID: 34351622

[91] Muddasani S Lin G Hooper J Sloan SB. Nutrition and nail disease. Clin Dermatol. (2021) 39:819–28. doi: 10.1016/j.clindermatol.2021.05.009 34785009

[92] Nahmani L Fitoussi F. Congenital nail abnormalities. Hand Surg Rehabil. (2024) 43:101527. doi: 10.1016/j.hansur.2023.01.011, PMID: 38879228

[93] Zaiac MN Walker A. Nail abnormalities associated with systemic pathologies. Clin Dermatol. (2013) 31:627–49. doi: 10.1016/j.clindermatol.2013.06.018, PMID: 24079592

[94] Gupta AK Nakrieko K-A. Trichophyton rubrum DNA strains in patients with onychomycosis with persistent mixed infections involving a nondermatophyte Mold. J Am Podiatr Med Assoc. (2020) 110:Article_3. doi: 10.7547/17-201, PMID: 32810210

[95] Gupta AK Renaud HJ Quinlan EM Shear NH Piguet V. The growing problem of antifungal resistance in onychomycosis and other superficial mycoses. Am J Clin Dermatol. (2021) 22:149–57. doi: 10.1007/s40257-020-00580-6, PMID: 33354740

[96] Gupta AK Summerbell RC Venkataraman M Quinlan EM. Nondermatophyte mould onychomycosis. J Eur Acad Dermatol Venereol. (2021) 35:1628–41. doi: 10.1111/jdv.17240 33763903

[97] Lim SS Ohn J Mun J-H. Diagnosis of onychomycosis: from conventional techniques and Dermoscopy to artificial intelligence. Front Med (Lausanne). (2021) 8:637216. doi: 10.3389/fmed.2021.637216, PMID: 33937282 PMC8081953

[98] Tabara K Szewczyk AE Bienias W Wojciechowska A Pastuszka M Oszukowska M. Amorolfine vs. ciclopirox - lacquers for the treatment of onychomycosis. Postepy Dermatol Alergol. (2015) 32:40–5. doi: 10.5114/pdia.2014.40968, PMID: 25821426 PMC4360009

[99] Iozumi K Abe M Ito Y Uesugi T Onoduka T Kato I. Efficacy of long-term treatment with efinaconazole 10% solution in patients with onychomycosis, including severe cases: a multicenter, single-arm study. J Dermatol. (2019) 46:641–51. doi: 10.1111/1346-8138.14935, PMID: 31206779 PMC6771904

[100] Gupta AK Versteeg SG. Tavaborole - a treatment for onychomycosis of the toenails. Expert Rev Clin Pharmacol. (2016) 9:1145–52. doi: 10.1080/17512433.2016.1206467 27347905

[101] Kawai M. Characteristics and efficacy of two topical therapeutic agents for onychomycosis. Med Mycol J. (2019) 60:71–4. doi: 10.3314/mmj.19.006, PMID: 31474693

